# Justified defection is neither justified nor unjustified in indirect reciprocity

**DOI:** 10.1371/journal.pone.0235137

**Published:** 2020-06-30

**Authors:** Hitoshi Yamamoto, Takahisa Suzuki, Ryohei Umetani

**Affiliations:** 1 Faculty of Business Administration, Rissho University, Tokyo, Japan; 2 Graduate School of Business Administration, Rissho University, Tokyo, Japan; 3 College of Policy Studies, Tsuda University, Tokyo, Japan; Carlos III University of Madrid, SPAIN

## Abstract

Indirect reciprocity is one of the major mechanisms in the evolution of human cooperation. In indirect reciprocity, social norms with which individuals distinguish good people from bad people play essential roles. Despite extensive studies on the evolution of cooperation in indirect reciprocity, little is known about which social norms people actually adopt. Here we reveal what kind of norms are adopted by people in indirect reciprocal situations in daily life by using scenario-based experiments. The results showed that people evaluated “justified defection” as neither good nor bad and withheld their evaluation. Theoretically, social norms that evaluate justified defection as good are required for cooperation to be stable. However, the norm that people actually adopted deviates from the theoretical predictions. Our results indicate the necessity to reconsider the justification of “justified defection” in the evolution of cooperation.

## Introduction

Mutual cooperation in a competitive situation has been an essential driver in the development of human society, but its mechanism is a difficult puzzle to solve. Especially, indirect reciprocity [1–8] is a significant solution for the large-scale and highly flexible systems often observed in modern society. For indirect reciprocity to work, norms are needed that distinguish good people from bad people so that cooperation is selectively directed to good people. To distinguish a good person from a bad person, two types of information about him are required. One is information about his past helpfulness (first-order information), and the other is information about the helpfulness of his recipient (second-order information).

The simplest norm of indirect reciprocity is image-scoring [3,4], which refers only to first-order information. However, image-scoring is not evolutionarily stable because it is vulnerable to errors and invasion by perfect defectors [9]. Therefore, second-order information is needed to sustain cooperation in indirect reciprocity. The major norms that use second-order information consist of stern-judging [10,11], simple-standing [1,5,12], and shunning [13]. As for the second-order information, it is crucial to consider the assessment rule when the player’s recipient has a bad reputation because evaluating the action of the player to a good recipient is easy. Naturally, cooperation with and defection against the good should respectively be evaluated as good and bad.

In particular, defection against a bad recipient (called justified defection) is a key factor in establishing cooperation [5,6,12,14–16]. This is because the main reason for the vulnerability of image-scoring is that it cannot distinguish justified defections from unjustified ones [9]. Furthermore, eight norms that can stabilize cooperation identified from 4,096 candidate norms by theoretical approaches (called the “leading eight”) [17,18] have a common relevant feature—if a good donor defects against a bad recipient, the donor is evaluated as good. In this study, we focus on the evaluation of justified defection in real human behavior.

Despite numerous experiments on indirect reciprocity [19–24] having been conducted, it remains unclear how humans actually use second-order information. On one hand, a study [19] has reported that people tend to use only first-order information because people do not like to process complex information when they can assess others on the basis of less complex information. On the other hand, other studies [25,26] have shown that even if there is a cost associated with obtaining information, people make decisions on the basis of both first- and second-order information. Furthermore, an assessment rule that ignores actions against bad recipients has been proposed as an evolutionally stable norm for cooperation [27,28]. In addition, experimental evidence shows that the evaluations about bad people are more uncertain than those about good ones [29].

As mentioned above, there is a deviation between predictions from theories and experimental results about the assessment rule for the actions against bad recipients. In particular, the distributions of assessment rules toward bad recipients in humans are unknown. By analyzing how second-order information, which is theoretically required to sustain cooperation, is used by humans in evaluating others, the function of the omission of punishment and the justified defection in human society will be revealed.

Here, we design experiments to clarify the distribution of people’s assessment rules in indirect reciprocity. In the scenario-based experiments, two people appeared in the scenario. One was a donor who does or does not cooperate with his/her recipient. The other was the recipient who asks the donor for help. Participants were asked to rate the donor as good or bad by observing when the donor chose to cooperate or not with the recipient. The recipient is labeled either good or bad. Participants evaluate four scenes in the combination of donor behaviors and recipient labels. Since the purpose of this study is to investigate which norms people adopt in real society, we adopted scenario-based experiments rather than an economic experiment.

Most theoretical studies have adopted an assumption in which a single norm pervades throughout a population resulting in all individuals using the same norm. However this assumption clearly oversimplifies the reality. In recent years, researchers have tackled this assumption by developing a model in which various norms coexist in the population [30,31]. However, if any of the norms proposed in the theoretical studies are actually adopted by people, the assessment of the donor by observers should be widely distributed. For example, if there is a mixture of good and bad evaluations for “justified defections” (i.e., not cooperating with bad recipients), the variance in the evaluation of justified defections should increase. This study explores whether people’s norms remain within the norms proposed in previous theoretical studies or whether people adopt norms that are different from those assumed in theoretical research.

## Methods

We designed three scenario-based experiments to explore how humans evaluate the donor’s action using the first and second-order information in indirect reciprocity. Participants were recruited through a Japanese crowdsourcing service (Yahoo! Crowdsourcing). To broaden the generalizability of findings, each experiment had completely different participants. All experiments were approved by the ethics committee of Rissho University, application number 29–3 and 30–15. Informed consent was obtained from all subjects.

### Experiment 1

**Fig 1:**
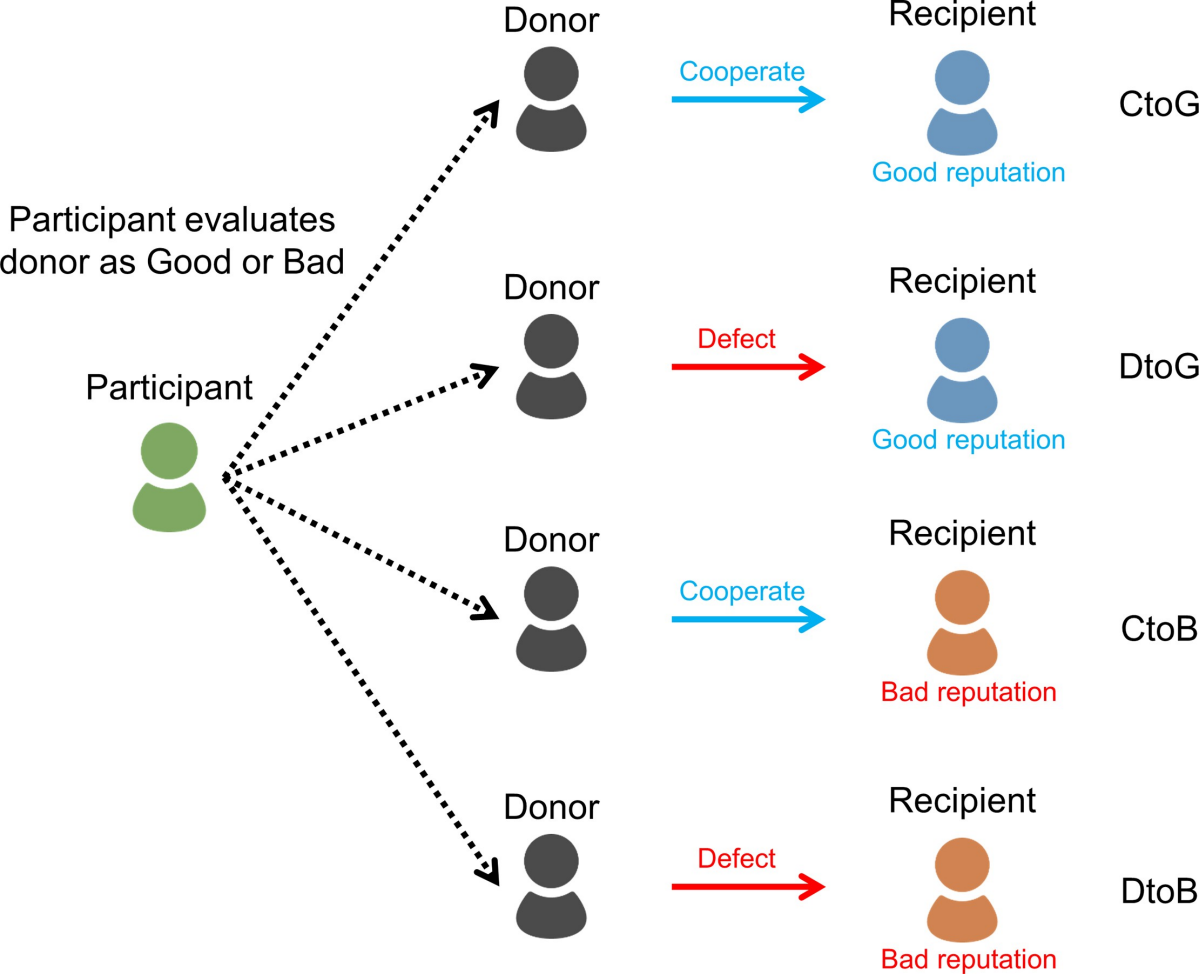
Overview of the experiment: Participants evaluate four scenes that consist of a combination of donor behavior (cooperate / defect) and recipient reputation (good / bad).

We recruited 200 participants (female = 71, mean age = 46.31 (S.D. = 9.96)). Participants evaluated a donor’s behavior using first- and second-order information. First-order information is the donor’s behavior (cooperation/defection), and second-order information is recipient reputation (good/bad). Combining both types of information, participants rated four scenes: cooperation with the good recipient (CtoG), defection against the good recipient (DtoG), cooperation with the bad recipient (CtoB), and defection against the bad recipient (DtoB) (Fig 1).

Participants first evaluated the scenes in order of CtoG and DtoG. To eliminate order effects on the cases of a bad recipient, the order of CtoB and DtoB was randomized among participants. The specific scenarios are as follows. In the first scenario, the participants were assumed to be workers in a restaurant. Assume that a colleague, Alice (recipient), asks another colleague, Bob (Donor), to take over the night shift, and Bob agrees (cooperation) or refuses (defection). We also controlled Alice’s reputation. The cases in which Alice has a good reputation are as follows.

Alice works hard and is always willing to take over when others cannot come to do the night shift. That is why Alice is liked very much by colleagues in the restaurant including you.

The cases in which Alice has a bad reputation are as follows.

Alice is not serious about her work and often takes time off from the night shift for various reasons. She often takes time off for selfish reasons and is not liked by her colleagues including you.

In the actual experiment, Alice and Bob’s names were converted into common Japanese names. After reading the scenario, participants rated how they assessed Bob’s behavior from three viewpoints using a 5-point scale: “Bob is a reliable person”, “Do you like Bob?”, and “Do you feel sympathetic to Bob?” The evaluation scores for the donor’s action were obtained by simply adding together the scores of these three statements (See S1–S3 Tables).

### Experiment 2

Experiment 2 was conducted using different scenarios in order to generalize the results by eliminating the dependence on a specific scenario. We recruited 200 participants (female = 93, mean age = 45.71 (S.D. = 10.45)). The basic settings are the same as for Experiment 1. The differences from Experiment 1 are as follows. The donor and recipient were changed from restaurant workers to neighborhood residents, and the recipient (Alice) asks the donor (Bob) for personal advice about distress. The cases in which Alice's has a good reputation are as follows.

Alice is always willing to respond to other people's requests and give them advice in a friendly manner. For this reason, Alice is well-liked by her acquaintances, including you.

The cases in which Alice has a bad reputation are as follows.

Alice has a habit of talking negatively about other people behind their backs. Also, she always thinks that she is right and sometimes forces her opinions on others, so her acquaintances, including you, feel that Alice is somewhat unpleasant.

### Experiment 3

We set up the scene of a giving game to examine not only the evaluation of behaviors in daily life but also behavior in an economic experiment. We recruited 200 participants (female = 87, mean age = 43.37 (S.D. = 12.31)). Participants were given an explanation of the rules of a giving game, in which a donor and a recipient were randomly matched in an iterated game, and then asked to rate the behavior of the donor. Participants rated donor behavior (donate / not donate) in the giving game the same as in Experiments 1 and 2. Recipient reputation was controlled by how many times he/she chose to donate during his/her past five rounds as a donor (5 (good), 0 (bad)). To show the participants that it was an economic game in an anonymous situation, we described the donor and the recipient as Player A and Player B. The cases in which the recipient (Player B) has a good reputation are as follows.

Player A is now the "donor" and Player B is now the "recipient". Player B selected "donate" all five times when he/she was previously the donor.

The cases in which Player B has a bad reputation are as follows.

Player A is now the "donor" and Player B is now the "recipient". Player B selected "not donate" all five times when he/she was previously the donor.

## Results

**Fig 2:**
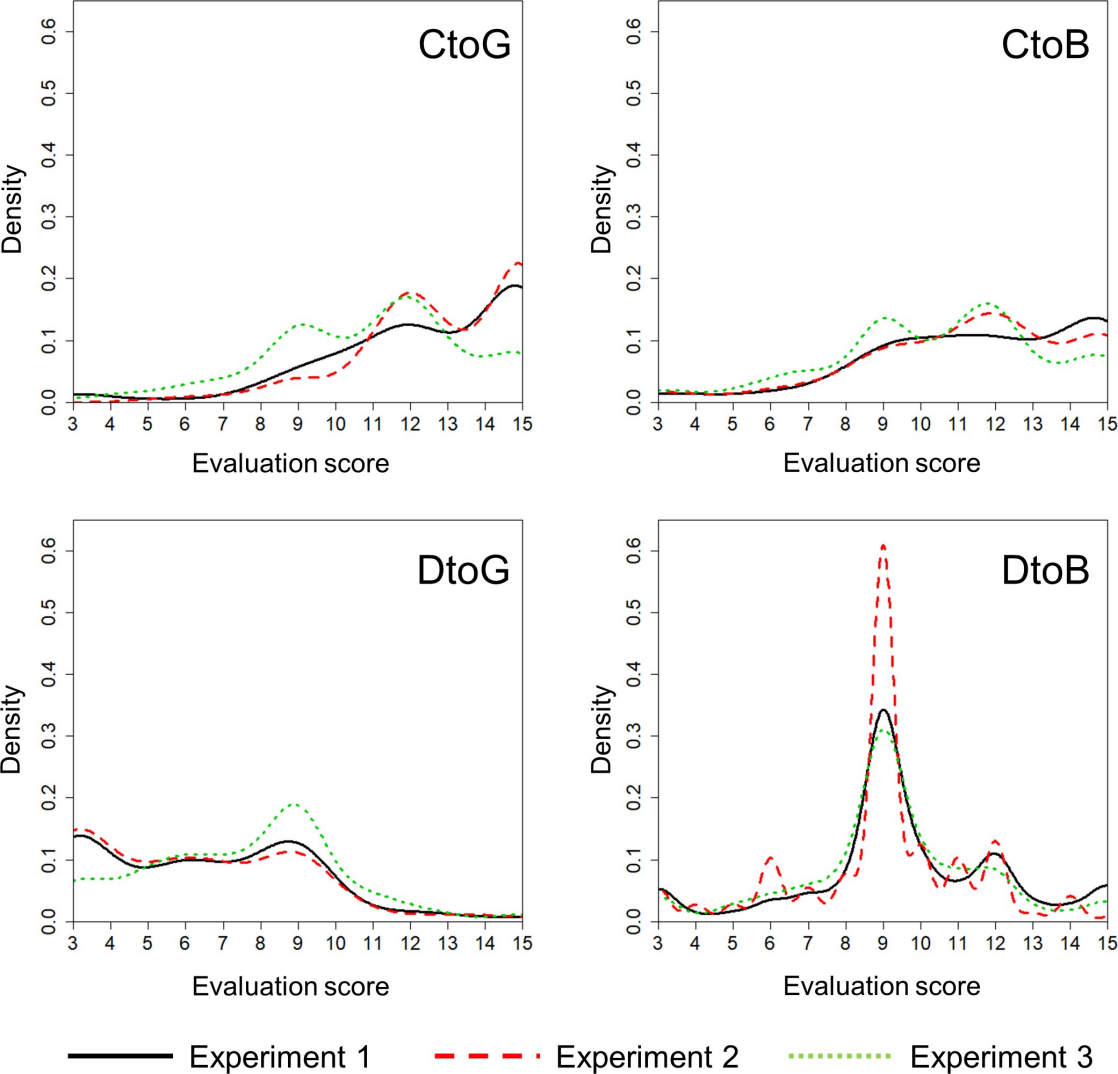
Distribution of evaluation for donors: The horizontal axis shows the evaluation score. The scores were calculated by simply adding together the scores of three statements on a 5-point scale, so it has minimum and maximum values of 3 and 15. The vertical axis shows the density of kernel density estimation. The solid black line, dotted red line, and dotted green line represent Experiments 1, 2, and 3, respectively.

We first analyze distributions of donor ratings in each scenario. The curves in all diagrams in Fig 2 are Gaussian kernel density estimations of the distributions of the evaluation of a donor’s action in each experiment. Kernel density estimation allows us to smooth over the distribution to avoid harsh edges caused by a sampling size, as found in the histogram method. Consistent with intuitive expectations, CtoG is rated as good and DtoG is rated as bad. CtoB is also rated as good. These results suggest that many participants adopt either image-scoring or simple-standing norms. DtoB, on the other hand, shows remarkable unique results. The distribution of ratings is centralized, and participants rate DtoB as neither good nor bad. If image-scoring and simple-standing are mixed in the participants, two peaks should appear, so it can be said that many participants do not adopt either image-scoring or simple-standing. In other words, participants adopt a norm of holding back on the evaluation to DtoB and not updating the reputation of donors.

To analyze these differences in distributions statistically, we employed quantile regression [32]. The ordinary linear regression assumes that the mean value represents the whole of the distribution and so enables us to estimate the effects of independent variables on the mean value of a dependent variable. The quantile regression enables us to estimate how independent variables affect each quantile of a dependent variable. Thus, the quantile regression could be used to explore the difference in the shape of the distribution of a dependent variable between conditions.

In this study, each quantile of evaluation scores was estimated for three experiments separately. To compare CtoB and other conditions, we used the CtoG dummy, DtoG dummy, and DtoB dummy as independent variables.

Table 1 shows the results of the quantile regression. The difference between CtoB and DtoB is small and not significant in the .25th quantile, but the .50th and .75th quantiles of CtoB are larger than those of DtoB, and these differences were significant. These results meant that the left half of the distribution was similar between CtoB and DtoB, and in another area, CtoB was more right-skewed than DtoB. The whole distribution of CtoB was similar to that of CtoG in that both were right-skewed and there was no significant difference at the 5 percent level, except the .25th and .50th quantiles of Experiment 2. DtoG is more left-skewed than CtoB in all experiments. To summarize these results, relative to CtoB, CtoG has a similar distribution, DtoG shifts to the left, and DtoB shifts to the center.

**Table 1:** Results of quantile regression: CtoB was the baseline category. We used “sqreg” command in Stata var.15 with a bootstrap method repeated 400 times.

		restaurant worker		personal advice		economic game	
		Coef.		Coef.		Coef.	
q25	CtoG	1		3	***	0	
	DtoB	-1		0		-1	
	DtoG	-7	***	-6	***	-3	***
	Cons	10	***	9	***	9	***
q50	CtoG	1		1	*	0	
	DtoB	-3	***	-3	***	-2	***
	DtoG	-6	***	-6	***	-3	***
	Cons	12	***	12	***	11	***
q75	CtoG	1		1		0	
	DtoB	-3	***	-4	***	-2	***
	DtoG	-5	***	-5	***	-3	***
	Cons	14	***	14	***	12	***

*p < .05.

**p < .01.

***p < .001.

## Discussion

For indirect reciprocity to work, people need to have norms that distinguish the good and bad of others’ behavior and cooperate only with good people. Extensive theoretical and empirical studies have looked for suitable norms for the evolution of cooperation, but there is no clear answer for the norms people adopt in our society. From a theoretical prediction, to stabilize cooperation through indirect reciprocity, a norm is needed that prescribes regarding as good those who do not cooperate with a bad person (justified defection) [5,6,12,14–16]. On the other hand, some empirical evidence has indicated that people use simple norms to rate cooperation as good and defection as bad [19]. We analyzed the distributions of norms in indirect reciprocity using scenario-based experiments.

Our results reveal that people do not evaluate the defection with a bad recipient as either good or bad and withheld their evaluation. This means that justified defection is neither justified nor unjustified. We also found, as expected, that cooperation was mostly considered good and defection with good recipients was considered bad.

As a result, the norms adopted by people can be summarized as shown in Table 2. Typical norms proposed by previous studies have unconditionally evaluated donor behavior as either good or bad. In fact, people held back on the evaluation to justifiably defect. The norm observed in our study, which can be described as GBGN (good, bad, good, neutral), is consistent with the “L1” rule of the “leading eight” norms [17] from the viewpoint of an evaluation rule. However, our experiments only examined the assessment rule and did not clarify behavioral rules [33]. The relationship between the evaluation rule and the behavioral rule is a future extension. It is necessary to explore why most participants adopt GBGN and under what conditions the norm is more advantageous than other norms.

**Table 2:** How social norms make assessments in indirect reciprocity: “G” and “B” describe a good and bad reputation, respectively. **“C” and “D” denote cooperation and defection, respectively.** “N” means the reputation of a donor remains neutral (“Neutral”).

Recipient’s reputation	G	G	B	B
Donor’s action	C	D	C	D
The observed norm	G	B	G	**N**
Simple-standing	G	B	G	G
Image-scoring	G	B	G	B
Stern-judging	G	B	B	G
Shunning	G	B	B	B

In a theoretical model of indirect reciprocity, a player’s reputation depends on his/her behavior, the reputation of his/her recipients, and the norms of observers. Therefore, the good and bad evaluations of the recipients may not be directly associated with cooperation and defection. To unify the reputations of the recipients among all participants in our experiments, they were designated as perfect cooperators or perfect defectors. In the future study, it will be necessary to consider the higher-order information, i.e. reputation of the donor or reputation of the previous recipient of the present recipient [34]. This may allow us to understand how the reputation of the recipients emerges under conditions of repeated interaction.

This paper is a first step to reveal a neutral reputation as an evaluation of justified defection. Many extensions are needed to understand the mechanism of this phenomenon. To explore the reason for the neutral evaluation of the defection against bad recipients, it is necessary to analyze whether the neutrality in evaluation is “don’t know” or “withhold evaluation.” People acquire an understanding of indirect reciprocity in childhood [35], so another challenging extension is to explore when second-order information begins to be adopted during growth. We should also consider cases where the observer has had a direct reciprocal relationship with the donor or recipient in the past [36]. Extensions by theoretical approaches can also be considered. It is necessary to analyze whether GBGN is evolutionally stable. Another extension would be exploring whether the GBGN norm survives in an environment where many norms coexist [30].

## Supporting information

S1 Fig Experiment 1. Restaurant worker scenario.
(DOCX)

S2 Fig Experiment 2. Personal advice scenario.
(DOCX)

S3 Fig Experiment 3. Economic game scenario.
(DOCX)

S1 Table Experiment 1. Restaurant worker scenario.
(DOCX)

S2 Table Experiment 2. Personal advice scenario.
(DOCX)

S3 Table Experiment 3. Economic game scenario.
(DOCX)

S1 Text (DOCX)
