# Supplementary material for: Justified defection is neither justified nor unjustified in indirect reciprocity
Source: PLoS One. 2020 Jun 30;15(6):e0235137. doi: 10.1371/journal.pone.0235137 (PMC7326222; doi:10.1371/journal.pone.0235137)
Supplement: S3 Fig — Economic game scenario. (DOCX) [file pone.0235137.s003.docx]

Figure S3: Experiment 3. Economic game scenario.
