# Supplementary material for: Justified defection is neither justified nor unjustified in indirect reciprocity
Source: PLoS One. 2020 Jun 30;15(6):e0235137. doi: 10.1371/journal.pone.0235137 (PMC7326222; doi:10.1371/journal.pone.0235137)
Supplement: S1 Table — Restaurant worker scenario. (DOCX) [file pone.0235137.s004.docx]

Table S1: Experiment 1. Restaurant worker scenario.

| **Scene** | **Mean** | **S.D.** | **Skewness** | **Kurtosis** | **α** |
| --- | --- | --- | --- | --- | --- |
| CtoG | 12.41 | 2.79 | -1.28 | 1.73 | 0.92 |
| DtoG | 6.44 | 2.91 | 0.46 | -0.36 | 0.89 |
| CtoB | 11.57 | 3.04 | -0.75 | 0.12 | 0.90 |
| DtoB | 9.49 | 2.80 | -0.23 | 0.39 | 0.93 |
