# Supplementary material for: Justified defection is neither justified nor unjustified in indirect reciprocity
Source: PLoS One. 2020 Jun 30;15(6):e0235137. doi: 10.1371/journal.pone.0235137 (PMC7326222; doi:10.1371/journal.pone.0235137)
Supplement: S2 Table — Personal advice scenario. (DOCX) [file pone.0235137.s005.docx]

Table S2: Experiment 2. Personal advice scenario.

| **Scene** | **Mean** | **S.D.** | **Skewness** | **Kurtosis** | **α** |
| --- | --- | --- | --- | --- | --- |
| CtoG | 12.81 | 2.20 | -0.93 | 0.60 | 0.89 |
| DtoG | 6.24 | 2.89 | 0.60 | -0.22 | 0.94 |
| CtoB | 11.36 | 3.00 | -0.77 | 0.30 | 0.92 |
| DtoB | 8.97 | 2.35 | -0.43 | 0.69 | 0.88 |
