# Supplementary material for: Justified defection is neither justified nor unjustified in indirect reciprocity
Source: PLoS One. 2020 Jun 30;15(6):e0235137. doi: 10.1371/journal.pone.0235137 (PMC7326222; doi:10.1371/journal.pone.0235137)
Supplement: S3 Table — Economic game scenario. (DOCX) [file pone.0235137.s006.docx]

Table S3: Experiment 3. Economic game scenario.

| **Scene** | **Mean** | **S.D.** | **Skewness** | **Kurtosis** | **α** |
| --- | --- | --- | --- | --- | --- |
| CtoG | 10.94 | 2.68 | -0.43 | -0.16 | 0.83 |
| DtoG | 7.55 | 2.67 | 0.18 | -0.08 | 0.85 |
| CtoB | 10.47 | 2.94 | -0.46 | -0.15 | 0.85 |
| DtoB | 9.17 | 2.56 | -0.20 | 0.66 | 0.88 |
