# Supplementary material for: Justified defection is neither justified nor unjustified in indirect reciprocity
Source: PLoS One. 2020 Jun 30;15(6):e0235137. doi: 10.1371/journal.pone.0235137 (PMC7326222; doi:10.1371/journal.pone.0235137)
Supplement: S1 Text — (DOCX) [file pone.0235137.s007.docx]

**Supplementary Information**

Justified defection is neither justified nor unjustified in indirect reciprocity

Hitoshi Yamamoto^1,3^, Takahisa Suzuki^2^, Ryohei Umetani^3^

1 Faculty of Business Administration, Rissho University, Tokyo, Japan

2 College of Policy Studies, Tsuda University, Tokyo, Japan

3 Graduate School of Business Administration, Rissho University, Tokyo, Japan

Corresponding author: Hitoshi Yamamoto

This file includes:
Supplementary Text S1
Supplementary Tables S1 – S3

Supplementary Figures S1 – S3

**Text S1**

The evaluation scores for donor’s action were obtained by simply adding together the scores for the following statement and questions using a 5-point scale (“Bob is a reliable person”, “Do you like Bob?”, and “Do you feel sympathetic to Bob?”). We conducted an exploratory factor analysis using a maximum likelihood method. The results clearly indicated a single factor structure in each scene. Tables S1 to S3 show the basic statistics of the scores. The values “α” in tables represent Cronbach's alpha of factor analysis. Figures S1 to S3 show a frequency distribution graph. The data supporting the findings of this study are stored in an OSF data package titled ‘Data of justified defection is neither justified nor unjustified in indirect reciprocity’ (Yamamoto, 2020), which can be accessed at the below link.

<https://doi.org/10.17605/OSF.IO/KFBNH>
